# BUB1B promotes extrahepatic cholangiocarcinoma progression via JNK/c-Jun pathways

**DOI:** 10.1038/s41419-020-03234-x

**Published:** 2021-01-11

**Authors:** Chen Yu Jiao, Qin Chao Feng, Chang Xian Li, Dong Wang, Sheng Han, Yao Dong Zhang, Wang Jie Jiang, Jiang Chang, Xuehao Wang, Xiang Cheng Li

**Affiliations:** 1 Key Laboratory of Liver Transplantation, Chinese Academy of Medical Sciences, Hepatobiliary Center, The First Affiliated Hospital of Nanjing Medical University, Nanjing, Jiangsu Province China; 2 Department of surgery, JiangYuan Hospital Affiliated to Jiangsu Institute of Nuclear Medicine, Wuxi, Jiangsu Province China

**Keywords:** Bile duct cancer, Bile duct cancer

## Abstract

Currently, the controversy regarding the expression profile and function of BUB1B in different malignancies still exist. In this project, we aimed to explore the role and molecular mechanism of BUB1B in the progression of extrahepatic cholangiocarcinoma (ECC). The expression levels of BUB1B in human ECC were evaluated by immunohistochemistry, western blot, and real-time PCR. The role and mechanism of BUB1B in CCA cell proliferation and invasion were investigated in both in vitro and in vivo functional studies. To indicate the clinical significance, a tissue microarray was performed on 113 ECC patients, followed by univariate and multivariate analyses. The expression of BUB1B was increased in both human CCA tissues and CCA cells. Results from loss-of-function and gain-of-function experiments suggested that the inhibition of BUB1B decreased the proliferation and invasiveness of CCA cells in vitro and in vivo, while overexpression of BUB1B achieved the opposite effect. Furthermore, the activation of c-Jun N-terminal kinase-c-Jun (JNK)-c-Jun pathway was regulated by BUB1B. BUB1B regulated the proliferation and invasiveness of CAA cells in a JNK-c-Jun-dependent manner. Clinically, ECC patients with BUB1B high expression had worse overall survival and recurrence-free survival than those with BUB1B low expression. Multivariate analysis identified that BUB1B was an independent predictor for postoperative recurrence and overall survival of ECC patients. In conclusion, BUB1B promoted ECC progression via JNK/c-Jun pathways. These findings suggested that BUB1B could be a potential therapeutic target and a biomarker for predicting prognosis for ECC patients.

## Introduction

Cholangiocarcinoma (CCA), described as a malignancy that arises from the epithelial cells of the bile duct, is the second most common primary hepatobiliary malignancy^1^. CCA can be divided into intrahepatic cholangiocarcinoma (ICC) or extrahepatic cholangiocarcinoma (ECC) according to the tumor location in the biliary tree. ECC is the most common CCA accounting for ~75% of all CCA^2^. Complete surgical resection remains the first choice for the treatment of CCA patients. However, the rates of resectability and the long-term outcome after these therapies are less than satisfactory because of the high post-surgical recurrence^3–5^. Therefore, a clearer understanding of CCA growth and metastasis mechanisms is urgently necessary to find the potential therapeutic targets for CCA.

BUB1B (BUB1 mitotic checkpoint serine/threonine kinase B) is a member of the spindle assembly checkpoint (SAC) protein family^6^. SAC prevents premature sister chromatid separation until all kinetochores are properly attached to the mitotic spindle during mitosis^7^. BUB1B plays a central role in SAC signaling and stable attachment of kinetochores to spindle microtubules^8,9^. Although Huang et al.^10^ reported that the structure of the kinase domain of BUB1B in *Drosophila melanogaster* folded into a conformation predicted to be catalytically active, more evidence showed that BUB1B, an inactive pseudokinase, interacts directly with Cdc20, BUB3, and MAD2, constituting the mitotic checkpoint complex, which inhibit the activity of the anaphase-promoting complex or cyclosome (APC/C)^7,11,12^. Accordingly, the function of BUB1B is to ensure proper chromosome segregation by suppressing the onset of anaphase by inhibiting APC/C activation^13^. Given the critical role of BUB1B in mitotic checkpoint signaling and chromosome congression, impairment in BUB1B and SAC often results in aneuploidy and chromosomal instability, which can contribute to an increase in cancer incidence^14–16^.

The relationship between BUB1B expression and specific human malignancies remains to be clarified. It has been reported that low expression of BUB1B contributed to the initiation and progression of colon adenocarcinoma^17,18^. However, a large number of reports have demonstrated that overexpression of BUB1B was associated with progression and recurrence of pancreatic ductal adenocarcinoma, prostate cancer, hepatocellular carcinoma, and some other cancers^19–21^. BUB1B can mediate anchorage-independent survival and growth, thereby facilitating lung adenocarcinoma dissemination during metastasis^22^. Moreover, reduction of BUB1B level or inhibition of BUB1B kinase activity in human cancer cells resulted in massive chromosome loss and apoptotic cell death^23^. The Cancer Genome Atlas (TCGA) database data showed that the expression of BUB1B in CCA tissues was upregulated. However, the biological function and molecular regulatory mechanism of BUB1B in CCA remain unclear. Therefore, it was worthwhile to further explore the roles and precise mechanisms of BUB1B in CCA.

In the current study, we aimed to investigate the roles and mechanisms of BUB1B in CCA and then explore its prognostic prediction value. We first detected the expression profile of BUB1B in CCA tissues and cell lines. Then, we investigated the functional role of BUB1B in cell proliferation and invasiveness of CCA and further explored the underlying mechanism. After that, the clinical significance of BUB1B in ECC patients was explored. Together, our findings suggested that BUB1B promoted CCA proliferation and invasiveness, as well as a candidate biomarker of prognostic prediction and a potential therapeutic target for ECC patients.

## Materials and methods

### Patients and tissue samples

From June, 2008 to May, 2017, 113 patients with ECC who had undergone routine surgical procedures at the First Affiliated Hospital of Nanjing Medical University, Nanjing China were included in the present study. Patients who received neoadjuvant treatment before primary surgery were excluded. All patients were examined routinely every 3–6 months during the first 5 years of follow-up and once a year thereafter. Overall survival (OS) time was defined as the period of time in months from operation to death. Recurrence-free survival time was defined as the period of time in months from operation to recurrence. To validate the clinical significance of BUB1B in CCA, a tissue microarray, as well as the staining of BUB1B, was performed on 113 ECC patients. A total of 29 ECC samples with matched para-tumor tissues were collected for detecting messenger RNA (mRNA) expression. Furthermore, we also detected the mRNA expression of BUB1B in 30 pairs of ICC samples. All CCA tissues were collected using protocols approved by the Ethics Committee of The First Affiliated Hospital of Nanjing Medical University, and written informed consent was obtained from every patient.

### Tissue microarray

To validate the clinical significance of BUB1B in ECC, a tissue microarray was performed. The details of tissue microarray have been described in our previous article^24^. The quantitation of immunostaining for BUB1B was completed by Quick-score (*Q*-score) based on the intensity and heterogeneity by two independent researchers who were blinded regarding patient details. The positive rates were scored as 0 point (0–5%), 1 point (6–35%), 2 points (36–70%), and 3 points (71–100%). The score of the staining intensity was presented as 0 point (none), 1 point (low), 2 points (medium), and 3 points (high). The *Q*-score was the product of heterogeneity and intensity. The expression was defined as high when the *Q*-score were >4.

### Cell culture and transfection

CCA cell lines (RBE, QBC939, HCCC9810, and HUCCT) and normal bile duct epithelial cell line (HiBEC) were obtained from the Cell Bank of the Chinese Academy of Science (Shanghai, China). These cell lines were cultured in Dulbecco’s modified Eagle’s medium (Gibco, USA) with 10% fetal bovine serum (Biological Industries, Israel), penicillin/streptomycin 100 U/ml at 37 °C in a 5% CO_2_ humidified incubator. The BUB1B-knockdown lentivirus was designed and produced by Polybrene (Obio Technology) using pLKD-CMV-G and PR-U6-shRNA vector. BUB1B overexpression plasmid was designed by Shanghai Genechem Co., Ltd (GV144 vector, *Xho*I/*Bam*HI enzyme cleavage, seq:

5′-GTCCGGACTCAGATCTCGAGCTATGGCGGCGGTGAAGAAGGAAGGGG-3′; R: 5′-TATCTAGATCCGGTGGATCCTCACTGAAAGAGCAAAGCCCCAGGACTAG-3′).

### Quantitative real-time PCR

The mRNA was extracted from tumor and para-tumor tissues or cultured cells using the TRIzol reagent (Invitrogen, China), and the complementary DNA (cDNA) was synthesized from RNA templates. The PCR primers for BUB1B were: forward, 5′- AAATGACCCTCTGGATGTTTGG-3′; reverse, 5′-GCATAAACGCCCTAATTTAAGCC-3′. Quantitative real-time PCR was performed using the Thermal Cycler Dice Detection System with the SYBR Premix ExTaqTM (Takara Inc. Japan) to detect the expression of the gene. Glyceraldehyde 3-phosphate dehydrogenase (GAPDH) was used as the endogenous control to which the data were normalized. Relative quantification of target gene expression was evaluated using the comparative cycle threshold (Ct) method. Mean ± SD was calculated from three independent experiments.

### Western blot

The protein extraction reagent kit (Beyotime, China) and 0.2% phenylmethanesulfonyl fluoride were used to obtain the proteins of cells and tissue samples. Western blotting was done with a modified version of a previous method^25^. Anti-BUB1B (1:1000, ab183496), anti-p-c-Jun (1:1000, ab32385), and anti-p-c-Jun N-terminal kinase (JNK) (1:1000, ab4821) were purchased from Abcam. The NIH ImageJ software (National Institutes of Health, Bethesda, MD) was used to make the results visible. GAPDH and α-tubulin were used as an internal loading control.

### Immunohistochemical staining

The expression of BUB1B, JNK, and c-Jun in CCA tissues was detected by immunohistochemical (IHC) staining. The details of IHC staining have been described in our previous article^25^. The images were acquired and quantified by light microscopy and NIS-Elements v4.0 software (Nikon, Tokyo, Japan).

### Transwell invasion assay

Transwell invasion assay was applied for detecting the role of BUB1B in CCA cell invasiveness. The 200 μl serum-free media containing 2 × 10^5^ CCA cells was transferred to the upper chamber. The lower chamber contained 10% serum-positive media used as a chemoattractant. After 48 h, cells migrated through the membrane and were fixed, stained, and quantified as described previously^26^.

### Wound-healing assay

CCA cells (5 × 10^5^) were placed in six-well plates with serum-positive media for 24 h, and then physically wounded with a sterile 1000 μl pipette tip, following by washed with phosphate-buffered saline (PBS) and incubated with serum-free media. Wound closure was monitored at the defined positions after 0 and 48 h of migration by light microscope. Areas were analyzed with the ImageJ software and the assays were carried out in three independent experiments.

### MTT cell proliferation assay

CCA cells were incubated in 96-well plates (~1 × 10^4^ cells per well) at 37 °C for 24–72 h. Each well was washed by PBS and incubated with 20 ml of MTT (3-(4, 5-dimethylthiazol-2-yl)-2, 5-diphenyltetrazolium bromide) and 180 ml of dimethyl sulfoxide for 2 h. Cellular viability was quantified by measuring the absorbance at 450 nm.

### EdU pulse-chase incorporation

EdU (5-ethynyl-2′-deoxyuridine) is a thymidine analog that can be used to label proliferating cells. We used the Cell-Light EdU DNA Cell Proliferation Kit (RiboBio, Shanghai, China) to detect the proliferation function of different CCA cells according to the manufacturer’s instructions. Cells were seeded in each well of 6-well plates, and the nucleic acids in all cells were stained with DAPI (4′,6-diamidino-2-phenylindole) dye. Images were acquired using a fluorescence microscope (Olympus FSX100).

### Flow cytometric analysis of cell cycle

CCA cells were cultured for 24 h in 6-well plates. A total of 1 × 10^6^ cells were harvested and detected using a Cell Cycle Staining Kit (MultiSciences, Lianke Biotechnology Co., Ltd., China) for cell cycle analysis according to the manufacturer’s instructions. BD AccuriC6 flow cytometer (BD Biosciences, USA) was used to analyze the cell cycle.

### Colony formation assay

Two thousand cells were seeded into 60-mm culture dish and incubated for about 14 days at 37 °C to allow for colony formation. Colony immobilization was maintained with methyl alcohol for 30 min at −20 °C, and then crystal violet was used to stain and count colonies.

### Xenograft tumors model

BALB/C athymic nude mice (male, 4–6 weeks) were purchased from Keygen Biotech and randomly grouped. CCA cells (2 × 10^6^) transfected with lentivirus were injected subcutaneously into the flanks of mice to generate xenograft tumors. Xenografts in each group were observed periodically after injection. Nude mice were killed by cervical dislocation after 3 weeks and the xenografts were peeled off subcutaneously. The tumor volume was measured with a caliper and calculated in mm^3^ using the following formula: *V* = length × width^2^/2. The weight of the xenografts in each group was compared and used for further analysis. Animal experiments were performed in accordance with guidelines established by the Animal Center of Nanjing Medical University.

### Phosphokinase array

To further explore the mechanism of BUB1B in promoting CCA cell proliferation and invasiveness, the Phosphokinase Array Kit (ARY003B, R&D System, Minneapolis, MN) was employed to detect the relative levels of phosphorylation of 43 kinases. Cells were collected and lysed with a cell lysate buffer containing phosphatase and protease cocktail inhibitors (Roche, Mannheim, Germany), and then incubated at 4 °C for 15 min. After being centrifuged at 14,000 × *g* for 5 min, the supernatant was transferred into a clean test tube and the sample protein concentrations were quantified using the Bio-Rad Protein Assay Dye Reagent Concentrate (Bio-Rad, Hercules, CA) according to the manufacturer’s instructions. The Array Detection steps were followed according to the manufacturer’s protocol. The sample protein (600 μg) was incubated with an antibody-array membrane at 4 °C overnight. The signals were detected and quantified using an Image Quant™ Imager (GE Healthcare Bioscience AB, Uppsala, Sweden) after the membranes were incubated with cocktail-detection antibody and streptavidin horseradish peroxidase.

### JNK/c-Jun signaling validation

In order to validate whether BUB1B regulated proliferation and invasiveness of CAA cells in a JNK-c-Jun-dependent manner, the JNK activator anisomycin (MCE) was used to stimulate JNK/c-Jun pathway in BUB1B-knockdown cells. After BUB1B-knockdown cells adhered to the six-well plate, anisomycin was added into the well and incubated at 37 °C for 48 h.

### Statistical analysis

The significance of differences between groups was evaluated with Student’s *t* test and *χ*^2^ test as appropriate. Disease-free survival (DFS) and OS rates were calculated by the Kaplan–Meier method with the log-rank test applied for comparison. Univariate and multivariate survival and recurrence analyses were performed by Cox proportional hazard regression. Two-sided *p* values were calculated, and a probability level <0.05 was considered to be statistically significant. All statistical data were carried out using IBM SPSS software v20.0 (IBM Corp, Armonk, USA) and GraphPad 6.0 (GraphPad Software, CA).

## Results

### BUB1B was upregulated in CCA tissues and cell lines

**Fig. 1:**
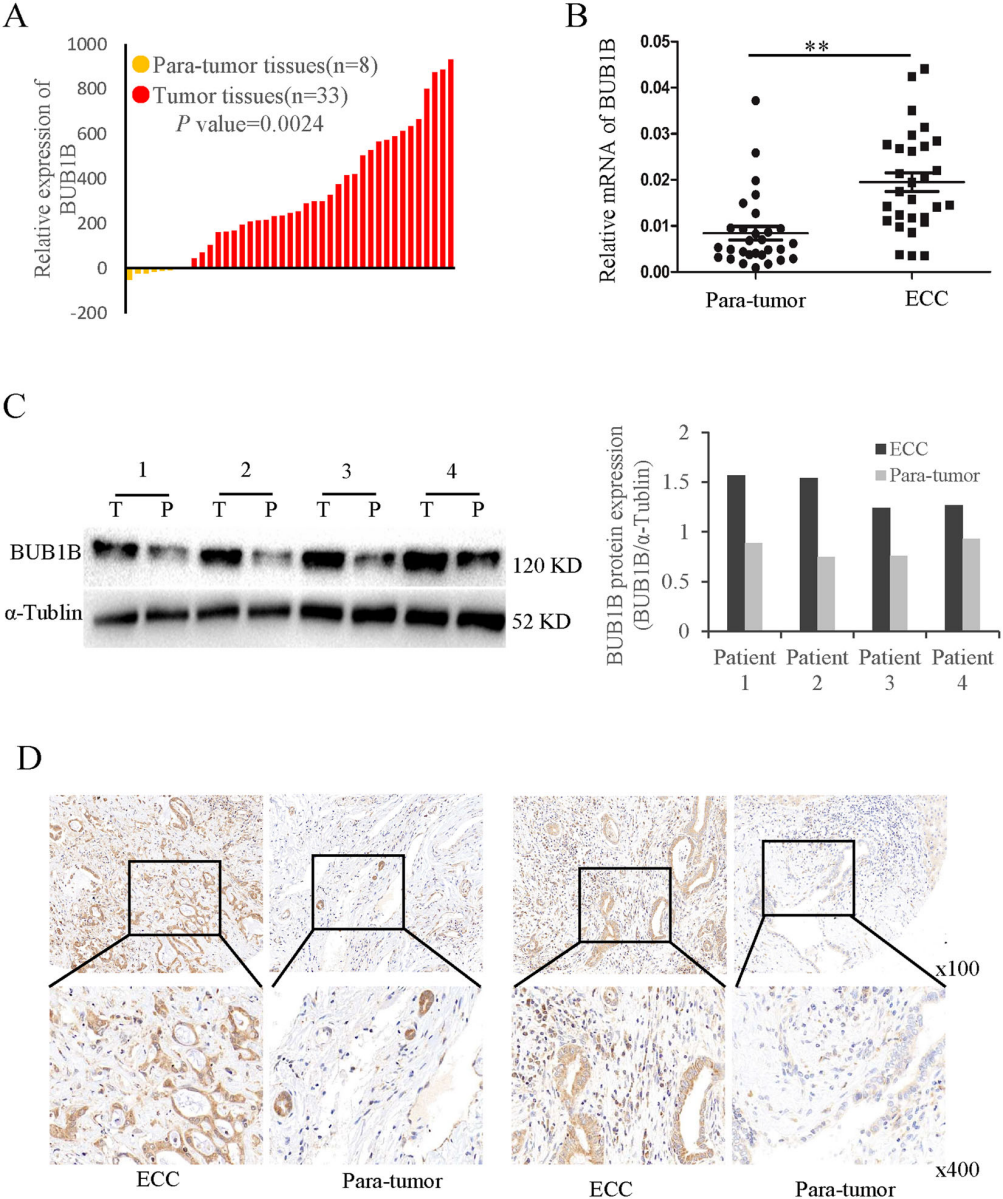
BUB1B was up-regulated in ECC tissues. **A** BUB1B was upregulated in CCA tumor tissues compared to paratumor in public TCGA database. **B** Our center samples also confirmed the expression mRNA level of BUB1B was upregulated in ECC tissues compared with their corresponding paratumor tissues. **C** The protein expression was determined for ECC samples by western blot. **D** BUB1B expression in the paired ECC samples was confirmed by IHC staining. The western blot and IHC staining illustrated that higher expression of BUB1B was found in ECC tissues (*p < 0.05, **p < 0.01).

To explore the expression profiles of BUB1B in CCA, we first examined the expression in CCA patients from a public TCGA database and found that BUB1B was overexpressed in CCA tissues compared to para-tumor (Fig. 1A). To further verify the data from public database, BUB1B mRNA levels of 29 paired ECC samples were measured by a quantitative reverse transcription-polymerase chain reaction. The results showed that the expression level of BUB1B was upregulated in ECC tissues compared with their corresponding para-tumor tissues (Fig. 1B). The western blot and IHC staining also illustrated that higher expression of BUB1B was found in ECC tissues (Fig. 1C, D). Similar to the expression file in ECC, the expression of BUB1B was also increased in ICC tissues with their corresponding para-tumor tissues (Supplementary Fig. 1A–D). Subsequently, we analyzed the expression profiles of BUB1B in CCA cell lines. The resulting data showed that compared to the normal bile duct cell line HiBEC, the expression of BUB1B was elevated in CCA cell lines, especially RBE and HCCC9810 (Supplementary Fig. 2A–C). Collectively, these results suggested that BUB1B expression was upregulated in both human CCA tissues and cell lines.

### BUB1B promoted the CCA cell proliferation and colony formation

**Fig. 2:**
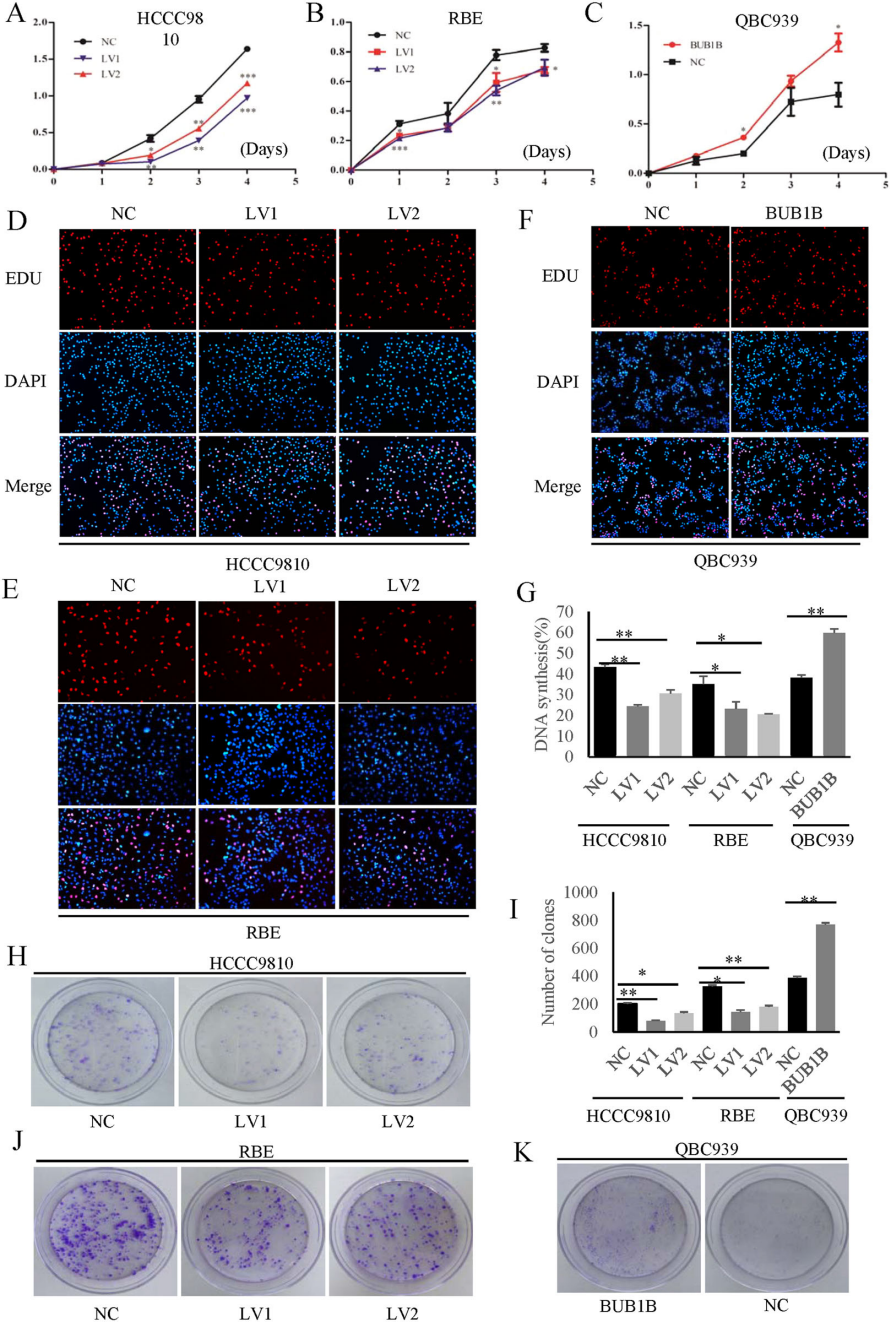
BUB1B promoted CCA cell proliferation. **A**–**C** Cell growth suppression was observed in knockdown group compared with control group in both HCCC9810 and RBE cells. In turn, the upregulated expression of BUB1B contributed to the proliferation of CCA cells. **D**–**G** The EdU staining demonstrated that the knockdown of BUB1B inhibited the proliferation of CCA cells while the over-expression of BUB1B promoted the cell proliferation. **H**–**K** The colony number and size were significantly lower in BUB1B knockdown group compared to control group. In contrast, the over-expression of BUB1B promoted the colony formation (*p < 0.05; **p < 0.01).

To determine the role of BUB1B in the proliferation of CCA cells, we used virus transfection to knockdown BUB1B in HCCC9810 and RBE cells and plasmid to overexpress BUB1B in QBC939 cells (with relatively low BUB1B level), respectively (Supplementary Fig. 3A–C). As expected, significant cell growth suppression was observed in the knockdown group compared with the control group in both HCCC9810 and RBE cells (Fig. 2A, B). In turn, the upregulated expression of BUB1B contributed to the proliferation of CCA cells (Fig. 2C). EdU staining also demonstrated that the knockdown of BUB1B substantially inhibited the proliferation of CCA cells, while the overexpression of BUB1B promoted cell proliferation (Fig. 2D–G). Furthermore, we also investigated the role of BUB1B in cell colony formation. The results showed that the colony number and size were significantly lower in the BUB1B-knockdown group compared to the control group. In contrast, the overexpression of BUB1B promoted colony formation (Fig. 2H–K). These data indicated that the expression of BUB1B was essential for maintaining cell proliferation and colony formation in CCA cells.

### BUB1B knockdown arrested the cell cycle at the G1/S phase in CCA cells

To further explore the mechanism of BUB1B promoted CCA cell proliferation, we performed flow cytometry to analyze the cell cycle status. The results showed that the fraction of CCA cells at the G1 phase was significantly higher, while the proportion in the S phase was remarkably decreased in the knockdown group compared with the control group (Supplementary Fig. 4A, B). Furthermore, the overexpression of BUB1B decreased the G1-phase proportion and increased the S-phase proportion (Supplementary Fig. 4C). These data demonstrated that BUB1B affected CAA cell proliferation by regulating cell cycle arrest.

### BUB1B promoted the invasiveness and tumorigenicity of CAA cells

**Fig. 3:**
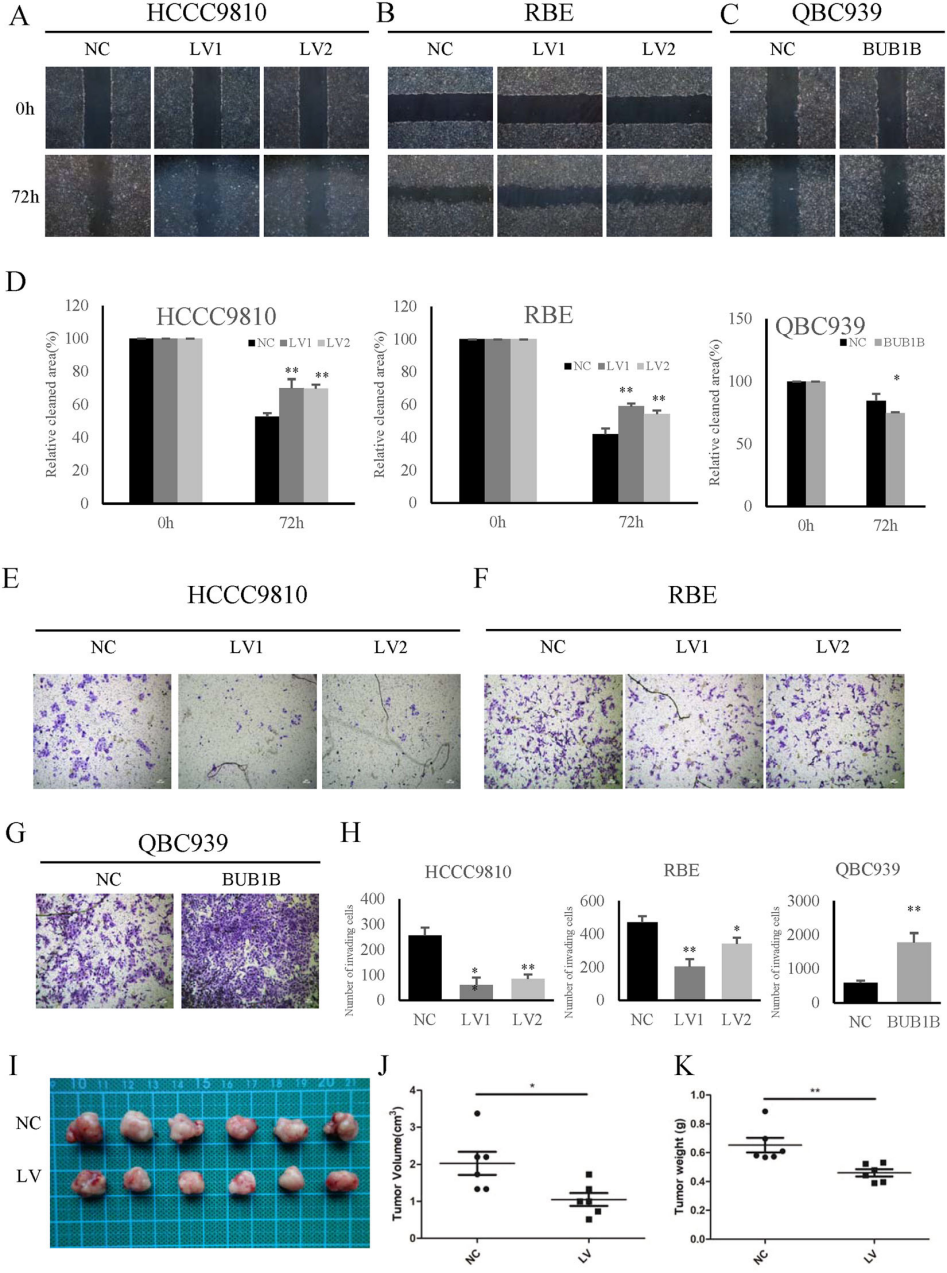
BUB1B promoted the invasiveness and tumorigenicity of CAA cells. **A**–**D** The knockdown of BUB1B inhibited the migration of CCA cells, while overexpression of BUB1B contributed to tumor cell migration in wound-healing assay. **E**–**H** In the transwell assay, the invasiveness of CCA cells was inhibited after knockdown of BUB1B and was enhanced in the overexpression group, respectively. **I**--**K** A subcutaneous xenograft tumor model in nude mice to determine the effect of BUB1B on tumorigenicity. The knockdown of BUB1B were able to suppress tumorigenicity resulting in obvious reduction in tumor volume (**J**) and weight (**K**) compared to the control group (*N* = 6) (*p < 0.05; **p < 0.01).

In order to explore the functional role of BUB1B in CCA, wound-healing and transwell assay were performed in the in vitro functional study. The results showed that the knockdown of BUB1B inhibited the migration of CCA cells, while overexpression of BUB1B contributed to tumor cell migration in wound-healing assay (Fig. 3A–D). Furthermore, in the transwell assay, the invasiveness of CCA cells was significantly inhibited after knockdown of BUB1B and was enhanced in the overexpression group, respectively (Fig. 3E–H). To further determine the effect of BUB1B on tumorigenicity, we performed a subcutaneous xenograft tumor model in nude mice. Our results showed that the knockdown of BUB1B was able to significantly suppress tumorigenicity, resulting in obvious reductions in tumor weight and volume compared to the control group (Fig. 3I–K). These data implied that BUB1B promoted CCA invasiveness and tumorigenicity.

### BUB1B regulated the expression of the JNK-c-Jun signaling

**Fig. 4:**
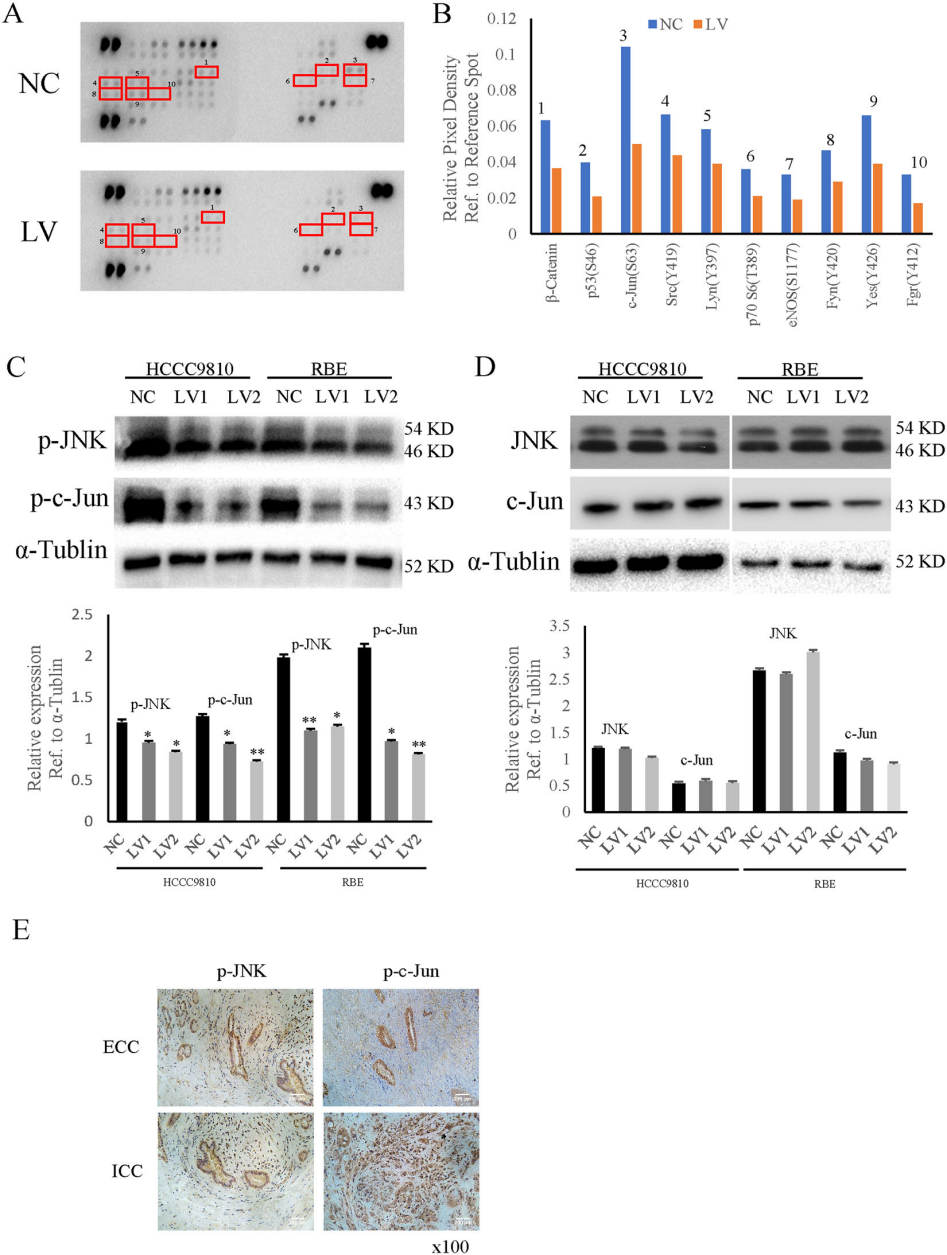
BUB1B regulated the expression of the JNK-c-Jun signaling. **A**, **B** Protein-chip analyses were performed. The knockdown of BUB1B suppressed the expressions of a series of proteins associated with tumor growth and invasiveness. **C**, **D** The expressions of JNK and c-Jun were detected by western blot. The expressions of phosphorylated c-Jun and JNK were inhibited in BUB1B-knockdown group. **E** The expressions of JNK and c-Jun in CCA were confirmed by IHC staining (*p < 0.05; **p < 0.01).

To further explore the mechanism of BUB1B in promoting CCA cell proliferation and invasiveness, protein-chip analyses were performed (Fig. 4A). The phosphorylated protein-chip results showed that the knockdown of BUB1B suppressed the expressions of a series of proteins associated with tumor growth and invasiveness (Fig. 4B). These results were further confirmed by western blot. The results showed that the knockdown of BUB1B suppressed the expression of phosphorylated c-Jun. We also found that the nuclear translocation of JNK (one of mitogen-activated protein kinases (MAPKs) was significantly inhibited in BUB1B-knockdown group (Fig. 4C). However, there were no obvious differences in Total JNK and c-Jun between the control group and the knockdown group (Fig. 4D). In addition, we further validated the results in the clinical samples of tumor tissues from ICC and ECC patients. IHC staining results demonstrated that the expression of phosphorylated c-Jun and JNK was also increased in ICC and ECC tissue samples (Fig. 4E). These data suggested that the JNK-c-Jun signaling could be regulated by BUB1B in CCA.

### BUB1B regulated proliferation and invasiveness of CAA cells in a JNK-c-Jun-dependent manner

**Fig. 5:**
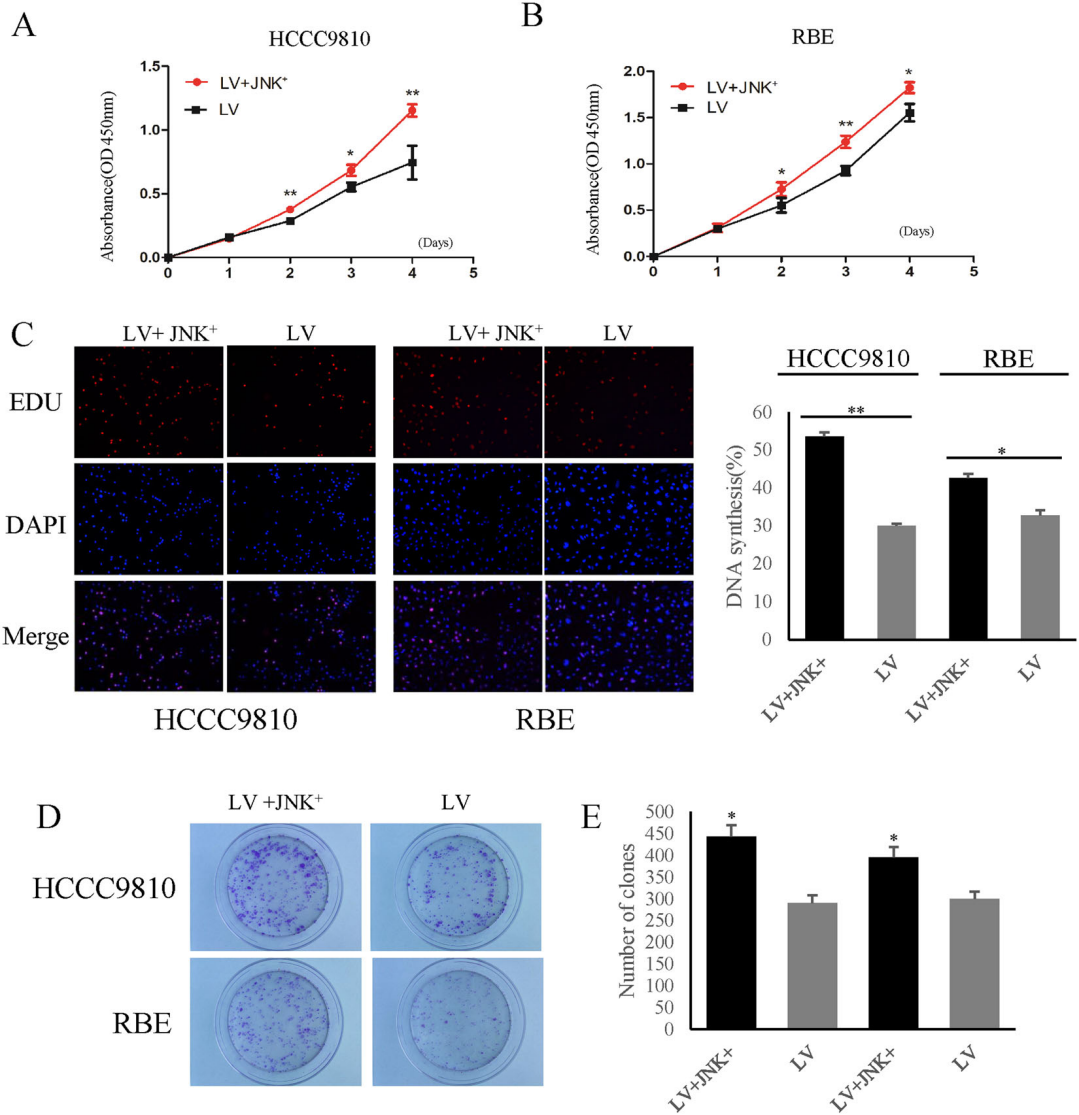
BUB1B regulated proliferation of CAA cells in a JNK-c-Jun-dependent manner. **A**, **B** CCA cell proliferation was detected by the MTT cell proliferation assay. The activation of JNK-c-Jun could largely rescue the inhibitory effect of knockdown BUB1B on proliferation in both HCCC9810 and RBE cells. **C** CCA cell proliferation was detected by EdU staining. **D**, **E** Representative images of colony formation assays and colony counts. The colony number and size were higher in the JNK-c-Jun activation group compared to the control group (*p < 0.05; **p < 0.01).

To determine whether JNK and c-Jun are key mediators of BUB1B’s function in cellular proliferation and invasiveness, we assessed the cells proliferation and invasiveness under the condition of BUB1B-knockdown and JNK-c-Jun activation. The JNK-c-Jun signaling was stimulated by anisomycin (Supplementary Fig. 5A, B). Compared to BUB1B-knockdown alone, the activation of JNK-c-Jun could largely rescue the inhibitory effect of knockdown BUB1B on proliferation in both HCCC9810 and RBE cells (Fig. 5A, B). The EdU staining also demonstrated that the activation of JNK-c-Jun promoted the cell proliferation of CCA cells (Fig. 5C). Furthermore, the results showed that the colony number and size were significantly higher in the JNK-c-Jun activation group compared to the control group (Fig. 5D, E).

**Fig. 6:**
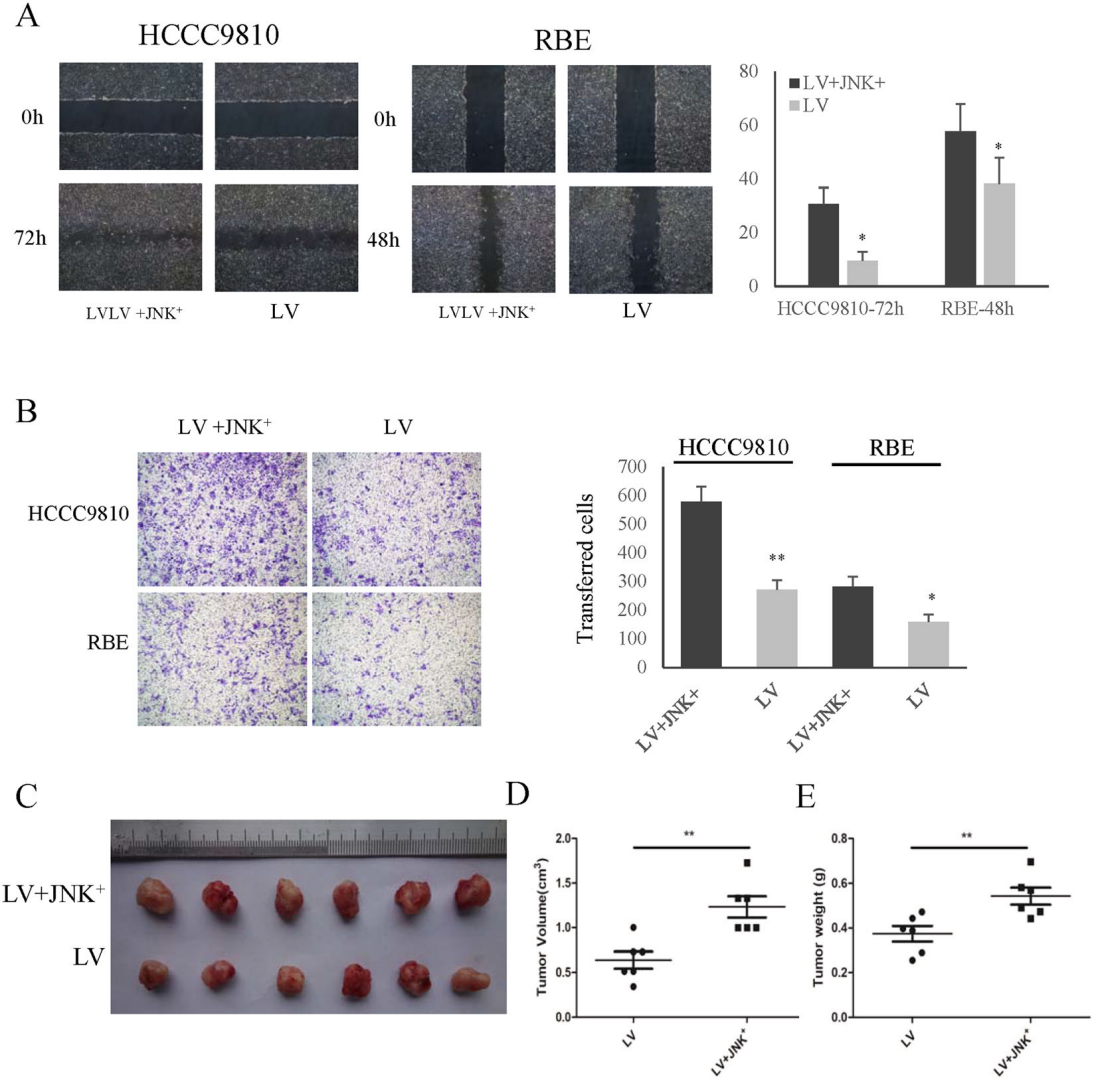
BUB1B regulated invasiveness of CAA cells in a JNK-c-Jun-dependent manner. **A** The ability of CCA cell invasiveness was enhanced after activation of JNK-c-Jun through wound healing. **B** Transwell assays showed that the activation of JNK-c-Jun promoted the CCA cell migration. **C**–**E** A subcutaneous xenograft tumor model in nude mice to determine the effect of BUB1B on tumorigenicity. The activation of JNK-c-Jun promoted tumorigenicity compared to the control group (*N* = 6) (*p < 0.05; **p < 0.01).

We next determined the effect of JNK-c-Jun activation on invasiveness and tumorigenicity in both in vivo and in vitro functional studies. The results showed that the ability of CCA cells’ invasiveness was enhanced after activation of JNK-c-Jun through wound healing (Fig. 6A). Moreover, the activation of JNK-c-Jun promoted CCA cell migration (Fig. 6B). The subcutaneous xenograft tumor model results also showed that the activation of JNK-c-Jun significantly promoted tumorigenicity compared to the control group (Fig. 6C–E). These results indicated that BUB1B promoted CCA cell proliferation and invasiveness through regulating JNK-c-Jun signaling.

### The clinical significance of BUB1B in ECC patients

**Fig. 7:**
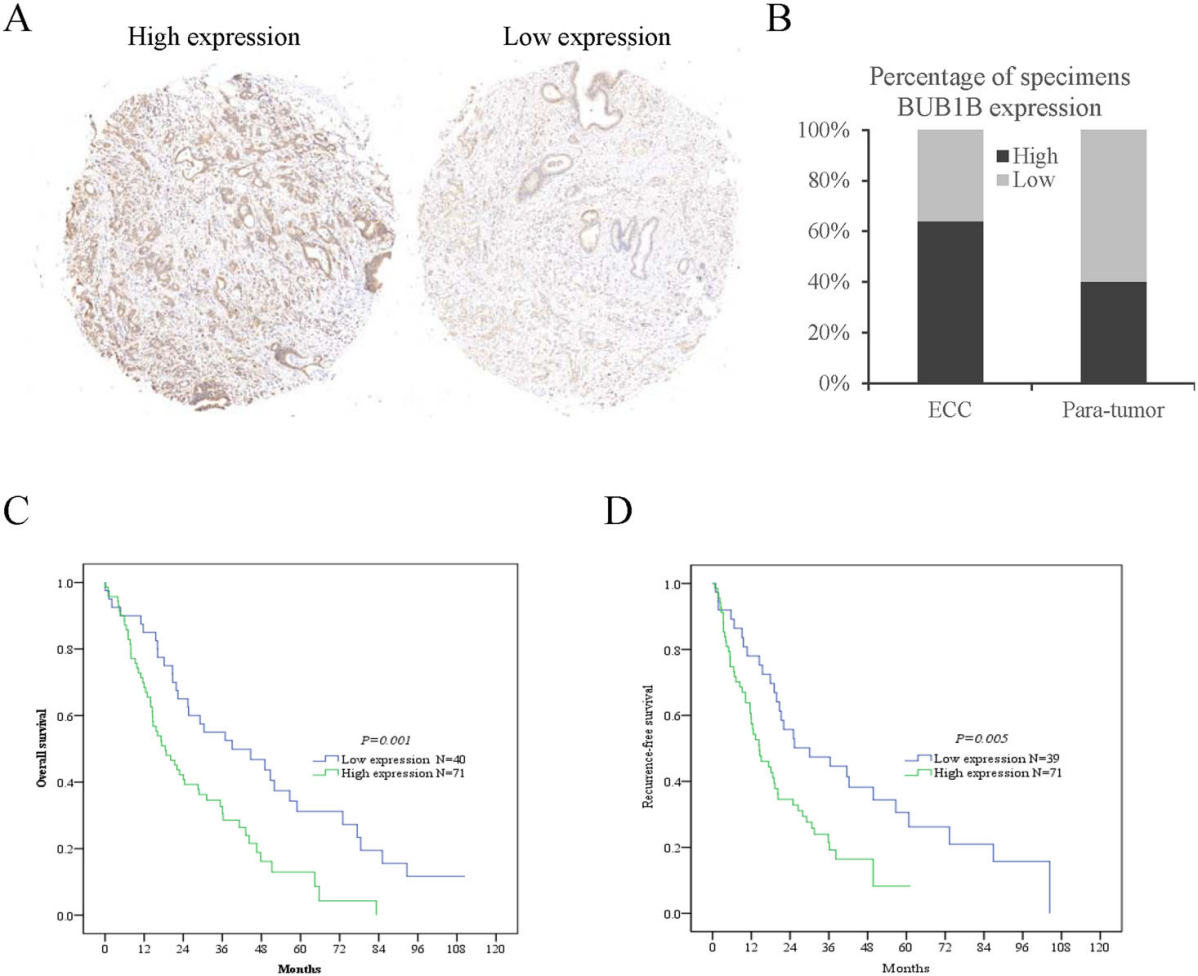
The clinical significance of BUB1B in ECC patients. ECC patients with BUB1B high expression had worse overall survival and recurrence-free survival than those with BUB1B low expression. **A**, **B** A tissue microarray and IHC staining were performed in 113 ECC patients. **C** Kaplan–Meier survival analyses were conducted to assess the influence of BUB1B on the overall survival of ECC. **D** Kaplan–Meier survival analyses were conducted to assess the influence of BUB1B on disease-free survival of ECC.

As a characteristic cancer-promoting factor, BUB1B promoted proliferation and invasiveness both in vivo and in vitro. To validate the clinical significance of BUB1B in ECC, a tissue microarray was performed on 113 ECC patients. According to the expression of BUB1B, the cohort of 113 ECC patients was divided into “BUB1B high” and “BUB1B low” expression groups (Fig. 7A, B). The clinicopathological parameters of these ECC patients and the correlation with BUB1B expression was described in Supplementary Table 1. Our results showed that there were no significant differences in expression and the clinicopathological parameters between the BUB1B high group and BUB1B low group. Kaplan–Meier survival analysis was used to determine whether the expression of BUB1B was associated with OS and recurrence-free survival of the ECC patients. The results showed that ECC patients with BUB1B high expression had worse OS and recurrence-free survival than those with BUB1B low expression (Fig. 7C, D). Furthermore, multivariate analysis identified that BUB1B was an independent predictor for postoperative recurrence and overall survival (Table 1). These results collectively suggested that up-regulation of BUB1B might have a stimulatory role in the progression and predict poor survival of ECC patients.

**Table 1:** Univariate and multivariate analyses for OS and recurrence-free survival of ECC patients.

Characteristic	OS		Recurrence-free survival	
	*P* value	HR (95% CI)	*P* value	HR (95% CI)
Univariate analysis				
Age (>60 vs. ≤60)	0.191	1.327 (0.868–2.029)	0.124	1.424 (0.907–2.236)
Sex (male vs. female)	0.325	0.801 (0.515–1.246)	0.043	0.61 (0.378–0.985)
CA19-9 (≥200 vs. <200 U/ml)	0.803	1.073 (0.615–1.875)	0.521	1.21 (0.676–2.166)
Tumor size (>3 vs. ≤3 cm)	0.719	1.091 (0.679–1.751)	0.873	1.042 (0.63–1.722)
Perineural invasion (yes vs. no)	0.031	1.677 (1.049–2.681)	0.106	1.494 (0.918–2.431)
Lymph node invasion (yes vs. no)	0.011	1.732 (1.137–2.637)	0.012	1.793 (1.137–2.828)
Tumor thrombus (yes vs. no)	0.044	2.003 (1.02–3.931)	0.658	1.193 (0.545–2.614)
TNM stage (III–IV vs. I–II)	0.066	1.531 (0.973–2.411)	0.009	1.881 (1.168–3.029)
R0 resection (yes vs. no)	0.012	1.951 (1.16–3.28)	0.011	2.004 (1.171–3.431)
BUB1B expression (high vs. low)	0.002	2.111 (1.322– 3.372)	0.005	2.036 (1.235–3.356)
Multivariate analysis				
BUB1B expression	0.003	2.163 (1.302–3.593)	0.002	2.374 (1.378–4.088)
R0 resection			0.011	2.070 (1.178–3.637)
Sex			0.01	0.501 (0.297–0.845)
Lymph node invasion	0.014	1.754 (1.120–2.748)		
TNM stage			0.01	1.961 (1.177–3.268)
Perineural invasion	0.047	1.677 (1.302–3.593)		

## Discussion

To our knowledge, this is the first report on the roles and mechanisms of BUB1B in CCA. In the present study, our results showed that the expression of BUB1B was upregulated in both CCA tissues and cell lines. It was consistent with the previous data displayed in the TCGA database. Furthermore, up-regulation of BUB1B was significantly correlated with poor OS and recurrence-free survival of ECC patients. The knockdown of BUB1B suppressed CCA cell proliferation, invasiveness, and tumorigenicity. Collectively, these results suggested that BUB1B could be a potential oncogene and competent prognostic marker of ECC.

The SAC prevents premature sister chromatid separation until all kinetochores are properly attached to the mitotic spindle during mitosis^7^. The BUB1B, as a member of the SAC protein family, plays important role in controlling mitotic timing in order to prevent the occurrence of aneuploid^27^. The abnormal expression and mutations of BUB1B can contribute to the development of cancer^28^. Currently, the controversy regarding the expression profile and function of BUB1B in different malignancies still exists. A large number of reports have demonstrated that overexpression of BUB1B was associated with progression and recurrence of bladder cancer, prostate cancer, hepatocellular carcinoma, and some other cancers^19–21^. Recent research showed that the up-regulation of BUB1B was associated with worse OS and DFS in pancreatic ductal adenocarcinoma and correlated with advanced tumor stage and tumor development^20^. Moreover, BUB1B can promote tumor proliferation and induce radioresistance in glioblastoma^29^. The reduction of BUB1B level or inhibition of BUB1B kinase activity in human cancer cells resulted in massive chromosome loss and apoptotic cell death^23^. Consistent with our present data, it was shown that BUB1B was overexpressed in ECC and promoted CCA cell proliferation and invasiveness. More importantly, BUB1B was significantly correlated with poor OS and recurrence-free survival of ECC patients. Therefore, BUB1B is a candidate oncogene for ECC risk prognostication and therapy. On the other hand, there are contradictory reports showing that BUB1B was dramatically reduced in colorectal adenocarcinomas and polyploid cells^17,18^. Colorectal carcinomas with low BUB1B expression were associated with frequent lymph node metastasis and poor prognosis^30^. The introduction of BUB1B triggered the apoptosis of polyploid cells formed by an aberrant exit from mitosis and inhibited the growth of tumors in athymic nude mice^17^.

JNK is a member of the MAPK protein family, which is involved in the control of cell proliferation, migration, and cancer progression^31,32^. c-Jun is activated through phosphorylation of serines 63 and 73, and then induces many transcription factors that are activated, such as ETS, NF-κB, and AP-1, which results in many important cell-proliferating and growth-regulating factors, such as c-Myc, cyclin D1, and c-Fos^33^. A recent study reported that the activation of JNK/c-Jun signaling played important roles in cholangiocellular proliferation, differentiation, and carcinogenesis^34^. Furthermore, the inhibition of JNK/c-Jun pathway by BAP1 suppressed intrahepatic CAA progression^35^. In this study, the results of the phosphokinase array indicated that JNK/c-Jun activation was positively correlated with the BUB1B expression in our research. The expressions of p-JNK and p-c-Jun were down-regulated in BUB1B-knockdown CCA cell lines. More interestingly, the activation of JNK-c-Jun could largely rescue the inhibitory effect of knockdown BUB1B on proliferation and invasiveness in CCA cells. Taken together, our research suggested that BUB1B positively regulated the JNK/c-Jun signaling pathway to exert its tumor-promoted functions in CCA. However, further research should be undertaken to investigate the precise mechanisms of the upstream of JNK/c-Jun regulated by BUB1B in CCA.

In summary, in the present study, we identified that BUB1B was upregulated in ECC and significantly correlated with poor OS and recurrence-free survival of the ECC patients. The knockdown of BUB1B decreased cell proliferation, migration, and tumorigenicity, while overexpression of BUB1B achieved the opposite effect both in vitro and in vivo. In addition, we also demonstrated that BUB1B promoted ECC progression by regulating JNK-c-Jun signaling pathway. Collectively, our findings provide new insight into the molecular pathogenesis of ECC, and BUB1B may be a candidate biomarker of prognostic prediction for CCA patients. We hoped that our study could provide evidence to explore the novel therapeutic strategy targeting ECC.

## Supplementary information

supplementary figure 1

supplementary figure 2

supplementary figure 3

supplementary figure 4

supplementary figure 5

supplementary figure legend

supplementary table
