# Supplementary figures and images for: BUB1B promotes extrahepatic cholangiocarcinoma progression via JNK/c-Jun pathways
Source: Cell Death Dis. 2021 Jan 11;12(1):63. doi: 10.1038/s41419-020-03234-x (PMC7801618; doi:10.1038/s41419-020-03234-x)

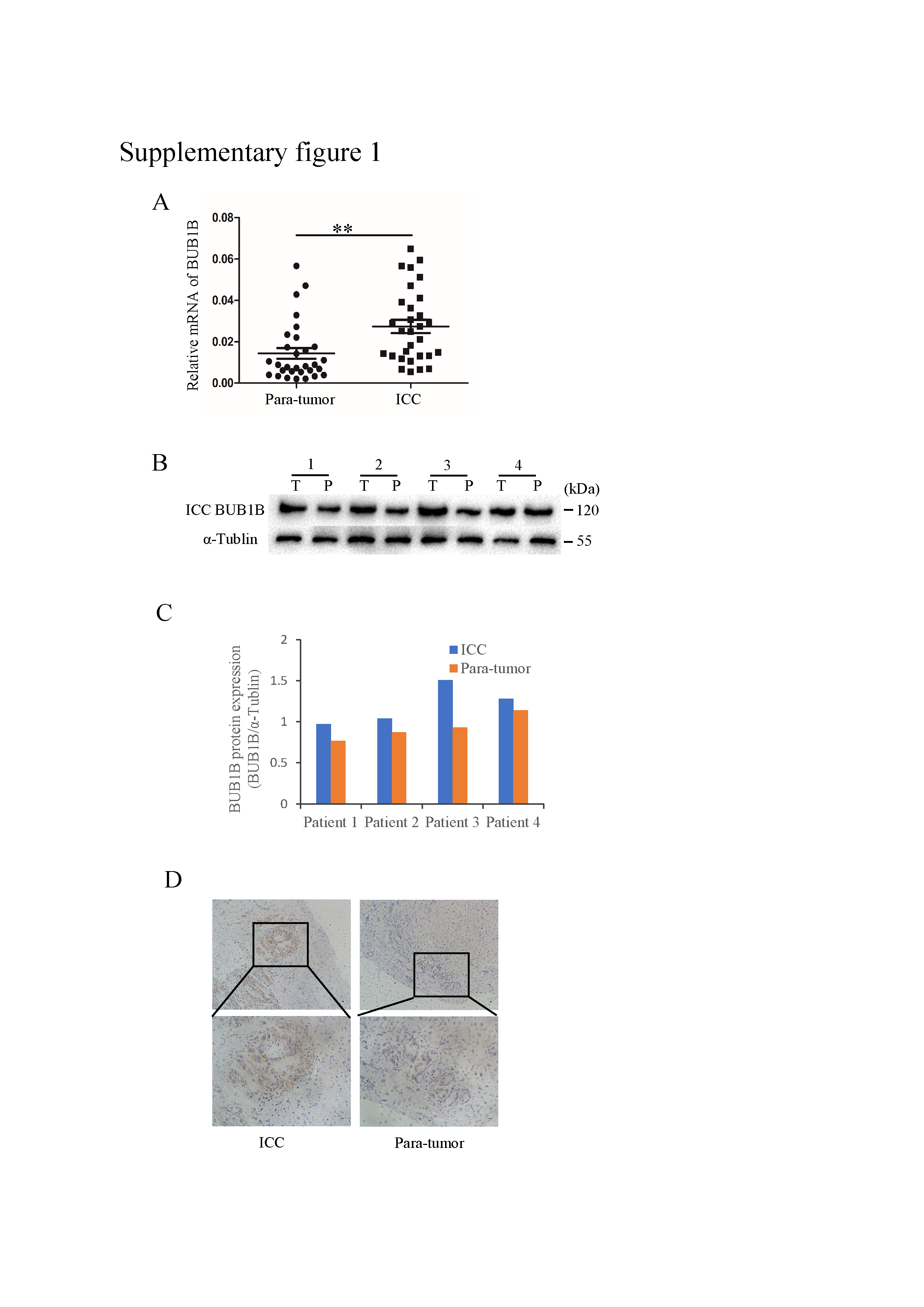

Supplement: Supplementary file 1 — supplementary figure 1 [file 41419_2020_3234_MOESM1_ESM.tif]

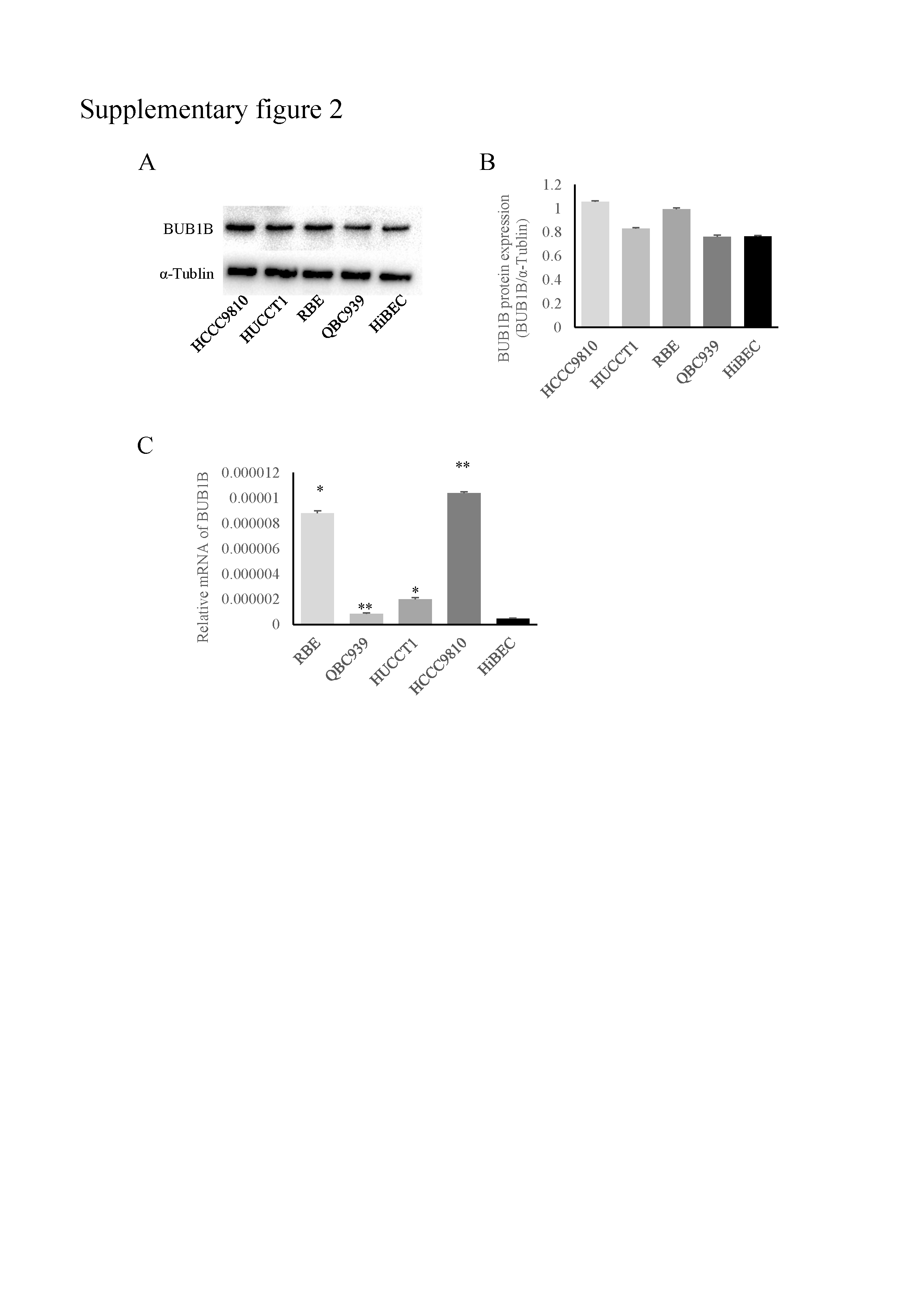

Supplement: Supplementary file 2 — supplementary figure 2 [file 41419_2020_3234_MOESM2_ESM.tif]

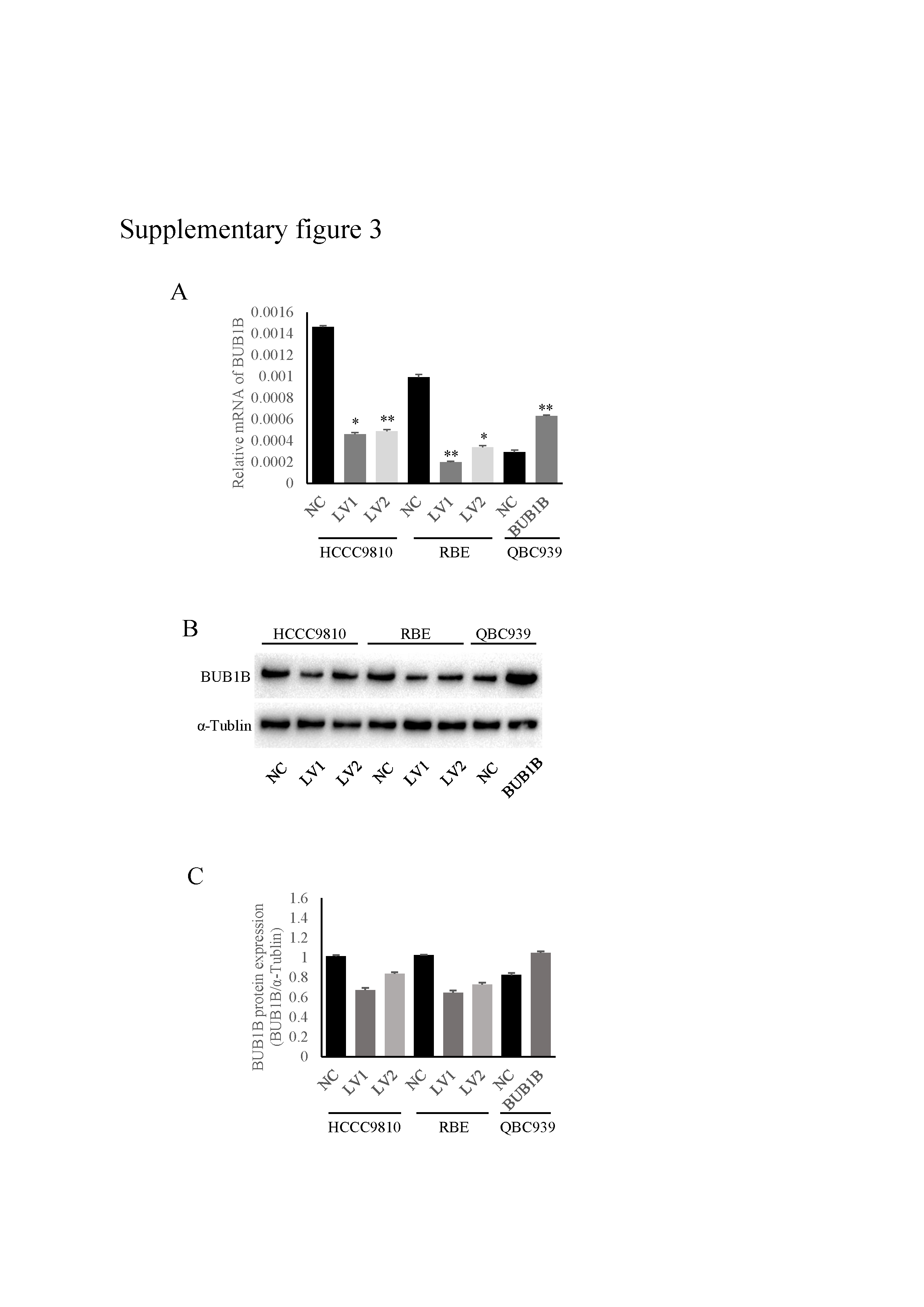

Supplement: Supplementary file 3 — supplementary figure 3 [file 41419_2020_3234_MOESM3_ESM.tif]

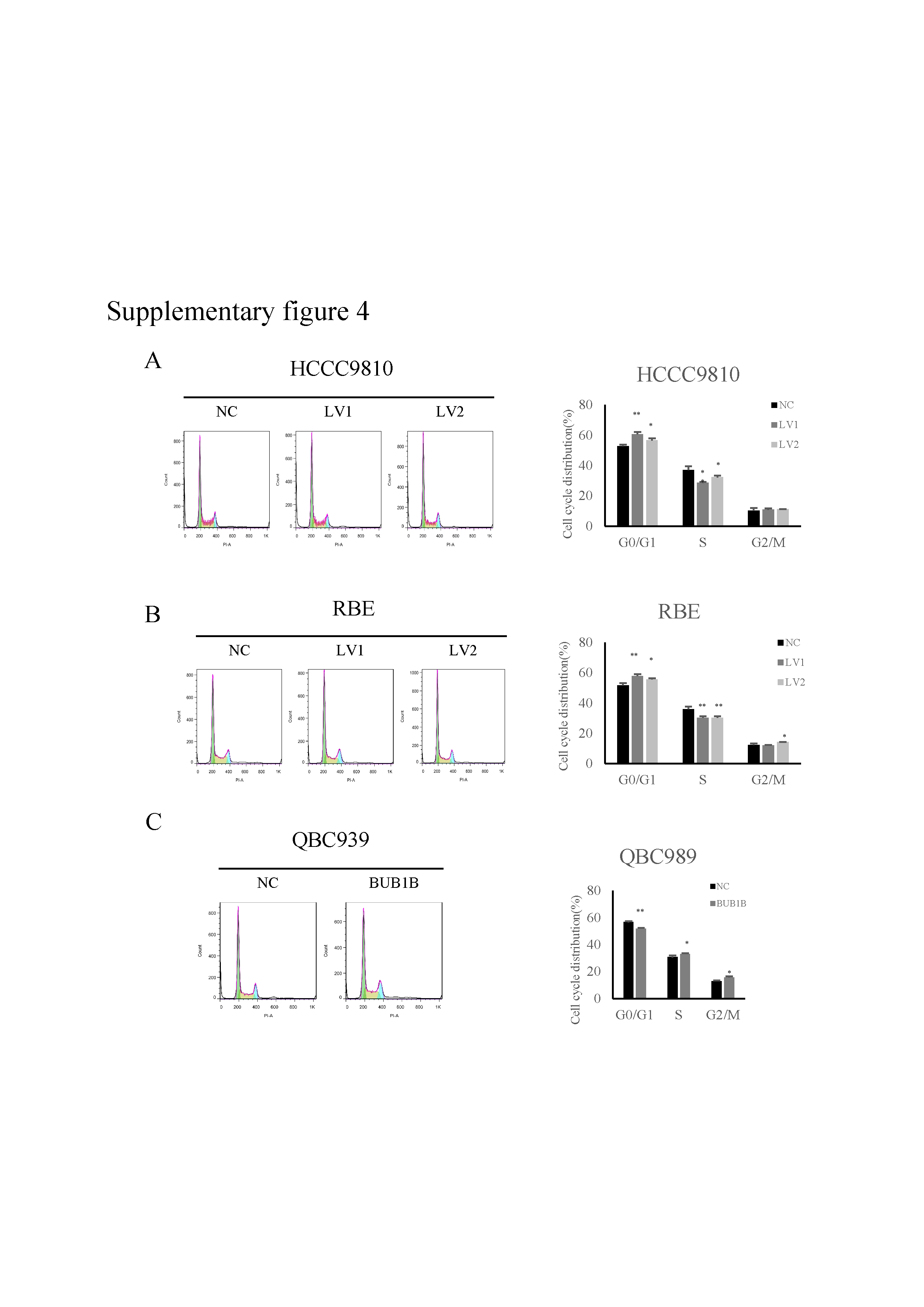

Supplement: Supplementary file 4 — supplementary figure 4 [file 41419_2020_3234_MOESM4_ESM.tif]

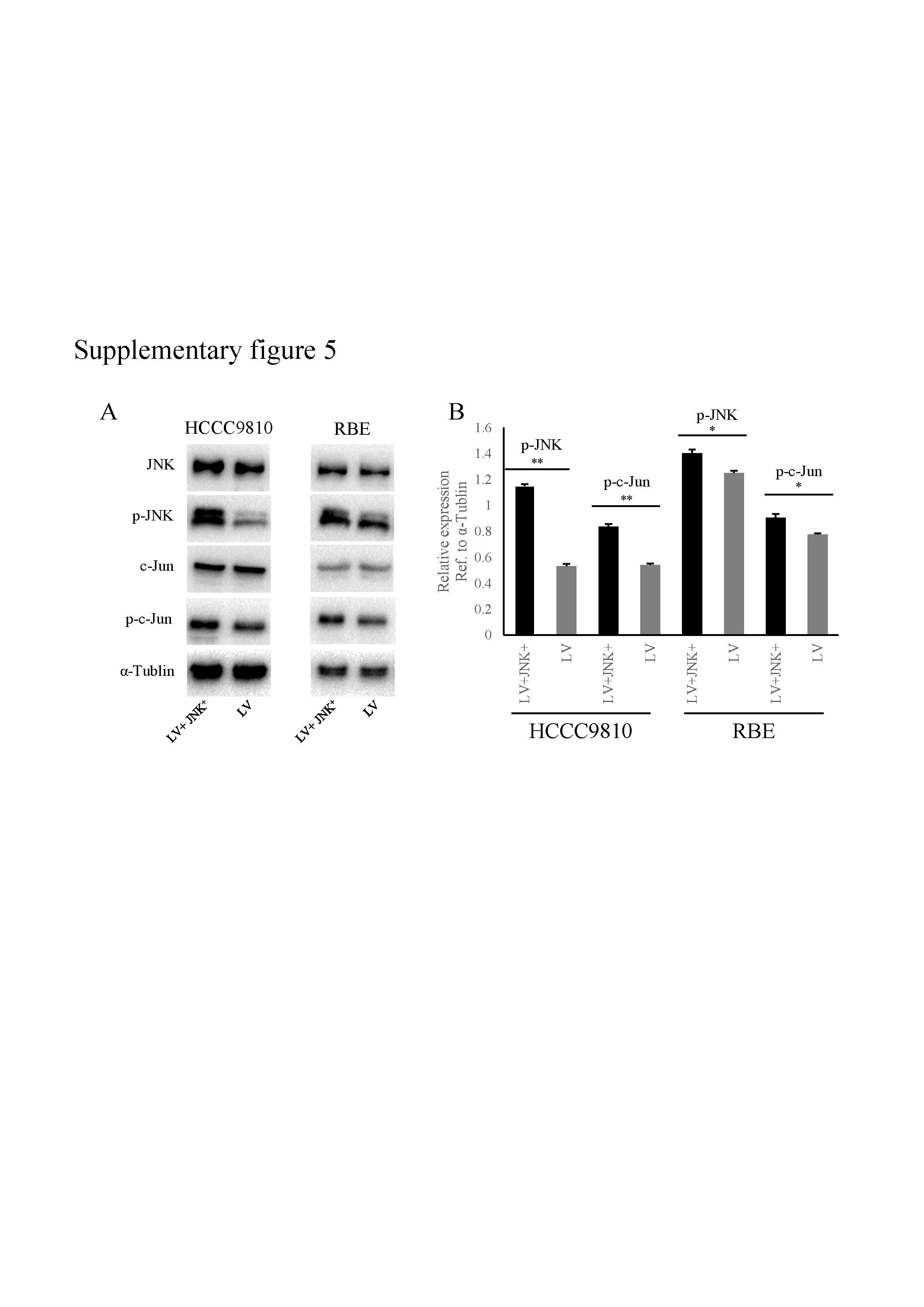

Supplement: Supplementary file 5 — supplementary figure 5 [file 41419_2020_3234_MOESM5_ESM.tif]
