# Supplementary material for: BUB1B promotes extrahepatic cholangiocarcinoma progression via JNK/c-Jun pathways
Source: Cell Death Dis. 2021 Jan 11;12(1):63. doi: 10.1038/s41419-020-03234-x (PMC7801618; doi:10.1038/s41419-020-03234-x)
Supplement: Supplementary file 6 — supplementary figure legend [file 41419_2020_3234_MOESM6_ESM.docx]

**Supplementary figure legends**

Supplementary figure 1. BUB1B was up-regulated in ICC samples. (A) The mRNA expression of BUB1B was determined for 30 pairs of ICC samples. (C) The protein expression was determined for ICC samples. (D) BUB1B expression in the paired

ICC samples was confirmed by IHC staining. *(﹡ p<0.0;﹡﹡ p<0.01)*

Supplementary figure 2. BUB1B was elevated in CCA cells compared to the normal bile duct cells line HiBEC. (A-B) The protein expression of BUB1B was determined for ICC samples by western blot. (C) The mRNA expression of BUB1B was determined in CCA cell lines by RT-PCR. *(﹡ p<0.0;﹡﹡ p<0.01)*

Supplementary figure 3. The down-regulated and up-regulated expression of BUB1B in cell lines. (A) The down-regulated and up-regulated mRNA expression of BUB1B was determined in CCA cell lines by RT-PCR. (B-C) The down-regulated and up-regulated protein expression of BUB1B was determined in CCA cell lines by western blot. *(﹡ p<0.0;﹡﹡ p<0.01)*

Supplementary figure 4. BUB1B knockdown arrested the cell cycle at G1/S phase in CCA cells. (A-C) The cell cycle status was detected by flow cytometry. *(﹡ p<0.0;﹡﹡ p<0.01)*

Supplementary figure 5. The JNK-c-Jun pathway was activated by anisomycin. (A-B) The activation of JNK-c-Jun pathway was detected by western blot.
