# Supplementary material for: BUB1B promotes extrahepatic cholangiocarcinoma progression via JNK/c-Jun pathways
Source: Cell Death Dis. 2021 Jan 11;12(1):63. doi: 10.1038/s41419-020-03234-x (PMC7801618; doi:10.1038/s41419-020-03234-x)
Supplement: Supplementary file 7 — supplementary table [file 41419_2020_3234_MOESM7_ESM.docx]

**Supplementary Materials**

Supplementary table 1. Clinicopathological relevance analysis of BUB1B expression in ECC patients

| Clinicopathological indexes | BUB1B | | |
| --- | --- | --- | --- |
|  | Low | High | P value |
| Age(year) |  |  | 0.285 |
| ≤60 | 15 | 35 |  |
| >60 | 25 | 38 |  |
| Sex |  |  | 0.192 |
| Male | 28 | 42 |  |
| Female | 12 | 31 |  |
| CA19-9 (U/mL) |  |  | 0.392 |
| <200 | 12 | 19 |  |
| ≥200 | 11 | 27 |  |
| Tumor size(cm) |  |  | 0.545 |
| ≤ 3 | 18 | 36 |  |
| >3 | 11 | 29 |  |
| Lymph node invasion |  |  | 0.667 |
| No | 23 | 45 |  |
| Yes | 17 | 28 |  |
| Perineural invasion |  |  | 0.125 |
| No | 16 | 19 |  |
| Yes | 24 | 54 |  |
| Tumor thrombus |  |  | 0.103 |
| Absent | 38 | 60 |  |
| Present | 2 | 13 |  |
| TNM stage |  |  | 0.225 |
| I + II | 11 | 29 |  |
| III + IV | 28 | 44 |  |
| R0 resection |  |  | 0.085 |
| Yes | 32 | 48 |  |
| No | 5 | 19 |  |
